# Formation of Natural Egg Yolk Granule Stabilized Pickering High Internal Phase Emulsions by Means of NaCl Ionic Strength and pH Change

**DOI:** 10.3390/foods11020229

**Published:** 2022-01-15

**Authors:** Sijie Mi, Minquan Xia, Xinyue Zhang, Jihong Liu, Zhaoxia Cai

**Affiliations:** 1 Hubei Hongshan Laboratory, National Research and Development Center for Egg Processing, College of Food Science and Technology, Huazhong Agricultural University, Wuhan 430070, China; misijie1997@163.com (S.M.); 13339732276@163.com (M.X.); 15972012639@163.com (X.Z.); 2 College of Science, Huazhong Agricultural University, Wuhan 430070, China; Jihong@mail.hzau.edu.cn

**Keywords:** egg yolk granule, Pickering high internal phase emulsions, partially hydrogenated oil substitutes, healthier diet

## Abstract

Pickering high internal phase emulsions (HIPEs) are gel-like concentrated emulsions that have the potential to be an alternative to partially hydrogenated oil (PHO). In this study, egg yolk granules (EYGs), natural complexes of protein and lipid isolated from egg yolk, were used as an emulsifier to prepare Pickering HIPEs. Gel-like HIPEs with an oil phase volume fraction of 85% and with an emulsifier concentration of only 0.5% could be prepared by using EYGs as an emulsifier. The EYGs were able to form stable HIPEs at NaCl ionic strengths over 0.2 M and at pH over 5.0 with NaCl ionic strength of 0.3 M. The EYGs, which could stabilize HIPEs, were easily to adsorb and cover the oil-water interface to form emulsion droplets with small particle size. In addition, interacting EYGs in the aqueous phase formed a continuous network structure, and the oil droplets packed closely, exhibiting high elasticity and shear thinning behavior. Furthermore, the formed HIPEs had suitable storage stability with no significant changes in appearance and microstructure after storage for 60 days. This work can transform traditional oils from liquid-like to solid-like by using EYGs to enrich food processing diversity and improve the storage stability of oils while reducing the intake of PHO and providing a healthier diet for consumers.

## 1. Introduction

Excessive intake of trans fatty acids will increase the risk of cardiovascular disease, diabetes, hypertension, and cancer [1]. The main source of trans fatty acids in the food industry is partially hydrogenated oil (PHO), which can result in fat hardening. The U.S. Food and Drug Administration has banned the addition of trans fatty acids to foods in 2018 because of their harmful effects on humans [2]. Therefore, there is an urgent need to find a healthy substitute for partially hydrogenated oils in the food industry. At present, high internal phase emulsions (HIPEs) can be used to convert liquid-like oil directly into solid-like oil without the addition of PHO to meet the health needs of consumers [3].

HIPEs are generally defined as emulsion systems with internal phase volume fractions exceeding 74%. The droplets of HIPEs are tightly packed and form a network structure, in which the oil droplets are trapped, resulting in the gel-like rheology, solid-like appearance, and excellent stability [4]. Emulsifiers for stabilizing HIPEs include low molecular inorganic particles (such as silicon dioxide and titanium dioxide), macromolecules (such as artificial polymers and proteins), and other Pickering particles [4,5]. HIPEs stabilized by low molecule inorganic particles are not suitable for the food industry due to their high cost, high dosage, environmental pollution, and food safety [6]. However, HIPEs stabilized by food-derived Pickering particles have more advantages: less dosage required, better storage stability, high environmentally friendly degree (easy degradation), safety, and non-toxic side effects [7]. In addition, Pickering particles are irreversibly adsorbed to the oil-water interface, and the strong gravitational interaction between the particles also forms a rigid layer to protect the oil droplets [8]. Therefore, emulsions stabilized by Pickering particles are highly resistant to agglomeration and Ostwald ripening [8]. At present, more and more studies have proved that protein, as a food-grade Pickering particle, could stabilize HIPEs, such as whey protein [9], zein [2], ovalbumin [10], and gelatin [5]. However, most Pickering protein particles could not stabilize HIPEs alone. They stabilized HIPEs by interacting with other co-stabilizers such as polysaccharides or polyphenols, which might lead to a more complicated preparation process.

Egg yolk granules (EYGs) are precipitated material obtained by centrifuging the egg yolk solution, natural complexes of protein and lipid, which have been shown to be a novel food-derived Pickering stabilizer [11]. EYGs contain high-density lipoproteins (HDLs), phosvitin, and low-density lipoproteins (LDLs) [11]. As an outstanding food material, due to low cholesterol, low fat, high nutritional value, and excellent emulsifying properties, EYGs had the potential to replace egg yolk in the preparation of mayonnaise, a typical high internal phase emulsion [12]. Therefore, EYGs might have the potential to be used to prepare HIPEs. However, the strong calcium phosphate bridges structure of EYGs led to its low solubility, which in turn affected its emulsifying properties [13,14]. Fortunately, its dense structure caused by calcium phosphate bridges was highly regulated by pH and NaCl ionic strength [13]. Thus, EYGs with high solubility are possible to be obtained by adjusting pH and NaCl ionic strength in order to prepare emulsions with high oil content. Recent studies have confirmed that EYGs were able to stabilize 75% of the oil, and the formed emulsions were gel-like, suggesting that EYGs could be used to stabilize HIPEs [15]. However, the preparation of stable Pickering emulsions by means of EYGs and the role of EYGs in stabilizing them are still not clear.

It is well known that pH and NaCl ionic strength play a vital role in the formation of emulsions. The properties of EYGs were also highly dependent on pH and NaCl ionic strength of the surroundings. Therefore, the aim of this study was to obtain different morphologies of EYGs by modulating factors of pH and NaCl ionic strength to form HIPEs with excellent properties. In this paper, the physicochemical and morphological properties of EYGs were characterized in different pH and NaCl ionic strength, and their ability to form and stabilize HIPEs were investigated. Furthermore, the effect of oil phase volume fraction (φ) and EYGs concentration on the formation of HIPEs were studied. Finally, the storage stability of HIPEs stabilized by EYGs was assessed. These results have important significance for the replacement of PHO in the food industry.

## 2. Materials and Methods

### 2.1. Materials

All eggs used for the laboratory were purchased from Jiufeng Chicken Farm in Wuhan. Soybean oil was obtained from a local supermarket in Wuhan. Nile red and fluorescein isothiocyanate (FITC) were purchased from Sinopharm Chemical Reagent Co., Ltd. Other chemical reagents were bought from the Sigma Company and were analytical grade. All solutions were prepared in distilled water purified with Clever-S30 (Shanghai, China).

### 2.2. Preparation of EYGs

The method for preparing egg yolk granules referred to a previous study with some modifications [11]. Firstly, the shells were broken, and the liquid egg was placed in an egg yolk separator to remove most of the egg white. The egg yolk was then rolled on filter paper to remove the proteins from the surface of the egg yolk membrane. The egg yolk membrane was then poked through with tweezers to collect the egg yolk. Afterward, the collected egg yolk was added to an equal volume of 0.15 mol/L NaCl solution and stirred with a magnetic stirrer. The diluted egg yolk solution was then centrifuged at 10,000× *g* for 45 min at 4 °C. The precipitated material obtained was the EYGs. Later, the surface of granule precipitation was rinsed with distilled water and then collected from the bottles and kept in the freezer. Finally, the EYGs were completely freeze-dried for subsequent use. The apparatus model of the freeze drier was CTFD-10S. The temperature of the condenser was −70 °C, and the temperature of the shelf was from −20 to 20 °C at a speed of 4 °C/h.

### 2.3. EYGs Characterization

#### 2.3.1. Zeta Potential

The zeta potential of EYGs at different NaCl ionic strength (0, 0.1, 0.2, 0.3, and 0.5 M) and at a fixed NaCl ionic strength of 0.3 M with different pH (2.0, 3.0, 4.0, 5.0, 6.0, 7.0, 8.0, and 9.0) were determined by Zetasizer Nano ZS zeta potential analyzer (Malvern Instruments Ltd., Worcestershire, UK). The EYGs solutions were diluted to 0.1 mg/mL with distilled water. All the samples were examined three times at 25 °C.

#### 2.3.2. Scanning Electron Microscope (SEM)

SEM was carried out according to a previous study with some modifications [16]. JM-6390LV scanning electron microscope (Fukuyama, Japan) was used to observe the images of the EYGs. The EYGs were pasted to the tape adhering to the aluminum tube and then sprayed with gold before observation. Then, the images of the EYGs were observed.

### 2.4. Preparation of HIPEs Stabilized by EYGs

Pickering HIPEs were prepared using EYGs and soybean oil. Pickering HIPEs formed at different NaCl ionic strength (0, 0.1, 0.2, 0.3, and 0.5 M) or at a fixed NaCl ionic strength of 0.3 M with different pH (2.0, 3.0, 4.0, 5.0, 6.0, 7.0, 8.0, and 9.0). Pickering HIPEs were generated by a one-step homogenization method. The mixtures of soybean oil and the EYGs solutions at different φ (0.75–0.86) with different EYGs concentrations (0.4–3.0 wt%) were sheared at 12,000 rpm for 1 min.

### 2.5. Emulsion Characterization

#### 2.5.1. Particle Size Distribution of Emulsions

Mastersizer 2000 (Malvern Instruments Ltd., Malvern, Worcestershire, UK) were used to test droplet sizes of HIPEs according to a previous report [11]. The refractive indices of soybean oil and EYGs solutions were set to 1.467 and 1.330.

#### 2.5.2. Confocal Laser Scanning Microscope (CLSM)

FV12000MPE confocal laser scanning microscope (CLSM, Olympus Optical Co. Ltd., Tokyo, Japan) was used to observe the microstructure of oil droplets according to previous research with some modifications [17]. The proteins and oil droplets in the emulsion were stained with Nile red and FITC, respectively. Later, 10 µL of the emulsion was aspirated onto a slide and covered with a coverslip for subsequent observation.

#### 2.5.3. Optical Microscope

DM3000 optical microscope (Leica Instruments Ltd., Wetzlar, Germany) was also used to observe the microstructure of the emulsions. A total of 10 μL of the emulsion was aspirated onto a slide and covered with a coverslip. Then, the appropriate droplet image at 40 × magnification was searched.

#### 2.5.4. Rheological Properties of Emulsions

Rheometer (R2000ex, American TA Instruments, New York, NY, USA) was used to test storage modulus (G′) and loss modulus (G″) of the HIPEs according to a previous work with some modifications [18]. The plates used for measurement were 40 mm parallel plates, and the gap value was set to 1.0 mm. The range of oscillation strain was from 0.01 to 100%, at the frequency 1 Hz (strain sweeps). The range of frequency was from 0.1 to 10 Hz (frequency sweeps). The range of shear rate was from 1 to 100 1/s (apparent viscosity). The test temperature was 25 °C.

#### 2.5.5. Storage Stability of Emulsions

The storage stability of the HIPEs was tested after 60 days at 4 °C. A total of 0.02 wt% sodium azide was added to the emulsion to avoid the effects of microbial contamination. The appearance, droplet morphology, and rheological property of HIPEs before and after 60 days of storage were tested.

### 2.6. Statistic Analysis

All data were subjected to Duncan’s significance analysis at the 5% significance level by IBM SPSS Statistics 19.0 software (IBM 150, New York, NY, USA). All experiments were repeated three times. All the pictures were prepared through the Origin 2018 software.

## 3. Results and Discussion

### 3.1. Zeta Potential of EYGs at Different NaCl Ionic Strength and pH

**Figure 1:**
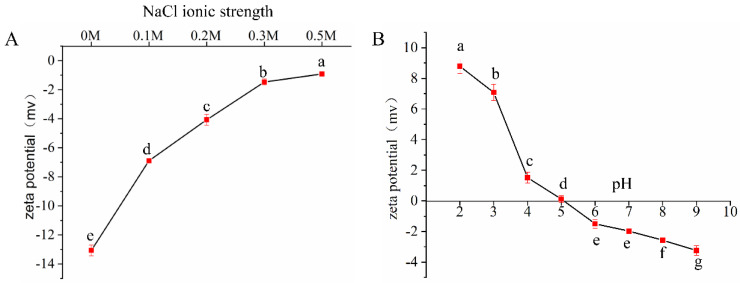
Zeta potential of EYGs at different NaCl ionic strength (**A**) and at a NaCl ionic strength of 0.3 M with different pH (**B**). Each value represents mean ± standard deviation (SD) of triplicates.

The zeta potential of the particles can indicate the surface charge of the particles. The surface charge of the particles plays an important role in the dispersion properties of the particles in aqueous solution. The zeta potential of EYGs at different NaCl ionic strength (0, 0.1, 0.2, 0.3, and 0.5 M) and at an NaCl ionic strength of 0.3 M under different pH (2.0, 3.0, 4.0, 5.0, 6.0, 7.0, 8.0, and 9.0) were shown in Figure 1. With the growing NaCl ionic strength from 0 to 0.5 M, the zeta potential of EYGs gradually increased from −13.07 mV to −0.92 mV (Figure 1A). The zeta potential of the EYGs was negative at NaCl ionic strength of 0 to 0.5 M, which might be related to the high content of phosphate groups [14]. The phenomenon of decreasing surface potential might be due to the electrostatic shielding effect of salt ions, which could reduce the surface charge of EYGs in solution, resulting in a reduction in electrostatic repulsion. Similar trends could also be observed in previous studies [19]. With the growing pH from 2.0 to 9.0, the zeta potential of EYGs gradually decreased from +8.79 mv to −3.24 mv (Figure 1B). The surface charge of the EYGs was positive under pH of 2.0 to 4.0, and it turned negative under pH of 6.0 to 9.0. As shown in Figure 1B, the isoelectric point of EYGs was close to 5.0. However, in the absence of NaCl salt ions, the isoelectric point of the EYGs was 4.0 instead of 5.0 [15]. Similarly, it had also been found that the isoelectric point of the EYGs was between 4.0 and 5.0 with the addition of 0.15 M NaCl in solution [20]. This result might be due to the increase in environmental NaCl ionic strength changing the ionization state of protein groups, which affected the number of surface charges. Therefore, the addition of NaCl to the solution could boost the isoelectric point of the EYGs. From pH 2.0 to 3.0, the absolute value of zeta potential of EYGs was much larger than that at other pH values, which might be due to the different aggregation states of EYGs under different pH. This phenomenon was consistent with the previous study [20].

### 3.2. Aggregation States of EYGs under Different NaCl Ionic Strength and pH

#### 3.2.1. Visual Appearance of Suspension

**Figure 2:**
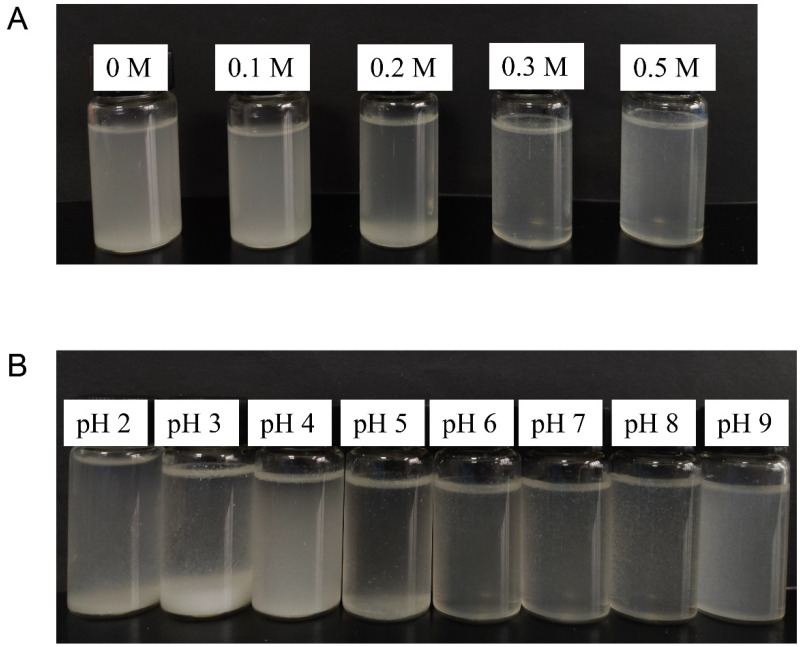
Visual appearance of suspension of EYGs at different NaCl ionic strength (**A**) and at a NaCl ionic strength of 0.3 M with different pH (**B**).

The visual appearance of particles in aqueous solution can reflect their aggregation states. The visual appearance of suspension of EYGs at different NaCl ionic strengths and at a NaCl ionic strength of 0.3 M with different pH is shown in Figure 2. As shown in Figure 2A, the suspension was turbid at NaCl ionic strengths of 0 and 0.1 M, while the NaCl ionic strength increased to 0.3 M, the suspension became clarified. This phenomenon implied that the EYGs in the suspension were changed from a larger aggregated state to a smaller dispersed state gradually with the continuous addition of NaCl. The turbidity of the suspension might also be related to the solubility of EYGs. At low NaCl ionic strength (<0.2 M), the main components of EYGs were HDL and phosvitin complexes connected by calcium phosphate bridges [14]. Because of these calcium phosphate bridges, the EYGs structure was very dense, which contributed to low solubility. At high NaCl ionic strength (>0.3 M), the divalent calcium of the calcium phosphate bridges of EYGs was replaced by monovalent sodium, resulting in the dissociation of the EYGs [21]. In the previous study, at a NaCl ionic strength below 0.1 M, the solubility of EYGs was low to 10% [13]. However, at NaCl ionic strengths over 0.3 M, their solubility increased to 80% and remained constant at 0.5 M because disrupted EYGs could release soluble phosvitin and HDL similar to soluble proteins [13,21].

As shown in Figure 2B, precipitation could be observed in the suspension at NaCl ionic strength of 0.3 M with pH 2.0, 3.0, and 4.0. While at pH over 4.0, the suspension became more clarified than that under low pH. This result was due to the fact that the EYGs were in the form of insoluble aggregates at low acidic pH and 0.3 M NaCl ionic strength, while the EYGs were in the form of soluble micelles at neutral pH and 0.3 M NaCl ionic strength [14]. In a previous study, Anton (2013) summarized that the protein solubility of EYGs was only 8% at a pH of 4.0 and a NaCl ionic strength of 0.1 M [14]. While the pH increased to 7.0, the protein solubility increased to 60% [14]. This further indicated that pH had a significant role in regulating the aggregation state of EYGs.

#### 3.2.2. SEM of EYGs

**Figure 3:**
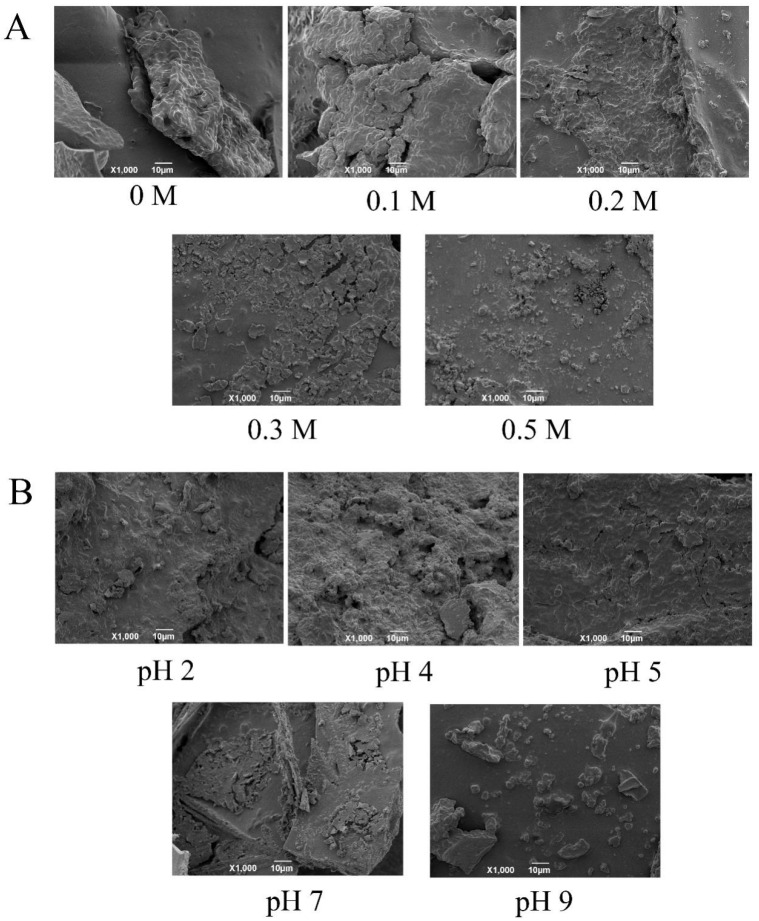
SEM image of EYGs at different NaCl ionic strength (**A**) and at a NaCl ionic strength of 0.3 M with different pH (**B**).

To observe the morphological change of EYGs at different NaCl ionic strengths and different pH, the SEM was employed. As shown in Figure 3, all the spherical particles were EYGs, which was similar to the description of EYGs in previous studies [22]. Moreover, the EYGs in the aggregation state could also be observed.

As shown in Figure 3A, EYGs were highly dense aggregated lumps without the addition of NaCl. With the NaCl ionic strength increased, the large aggregates of EYGs gradually dissociated into small and uniform micelles or aggregates, which was consistent with the results of the visual appearance of EYGs suspension (Figure 2A). Furthermore, in a recent study, the Z-average hydrodynamic diameter of EYGs decreased from 2646.1 ± 299.0 nm to 171.9 ± 3.1 nm as the NaCl ionic strength increased from 0 to 0.5 M at pH 6.0 [23].

As shown in Figure 3B, clustered aggregates of EYGs could be observed at pH of 2.0 and 4.0. While the aggregates of EYGs dissociated at pH over 5.0. These results were also consistent with the results of the visual appearance of suspension. These results might be due to that EYGs were complexes composed of LDL micelles and HDL particles, and the state of association among them varied with pH [13]. In addition, in previous studies, the Z-average hydrodynamic diameter of EYGs at pH 2.0, 3.0, and 4.0 was 2283.0 ± 420.7 nm, 3711.0 ± 111.7 nm, and 3646.0 ± 701.0 nm, respectively, at NaCl ionic strength of 0.3 M [23]. While the hydrodynamic diameter of EYGs decreased gradually from 1853.0 ± 207.5 nm to 108.3 ± 3.2 nm at pH from 5.0 to 9.0 with NaCl ionic strength of 0.3 M [23]. The results of the visual appearance of suspension and SEM suggested that the NaCl ionic strength and pH played an important role in the electrical charge and aggregation state of the EYGs. This knowledge may help to design HIPEs with different functions and properties.

### 3.3. Emulsion Characterization

Soybean oil (80% volume) was added to the EYGs solution to prepare HIPEs. In the pre-experiment, gel-like HIPEs could not be prepared only by changing the pH of the EYGs suspension (result not shown). However, by varying the NaCl ionic strength of the EYGs suspension, stable HIPE could be successfully prepared. Therefore, the effect of NaCl ionic strength on HIPE stabilized by EYGs was investigated, followed by the effect of pH on HIPEs stabilized by EYGs at a fixed NaCl ionic strength (0.3 M).

#### 3.3.1. Effect of NaCl Ionic Strength on Emulsion Properties

**Figure 4:**
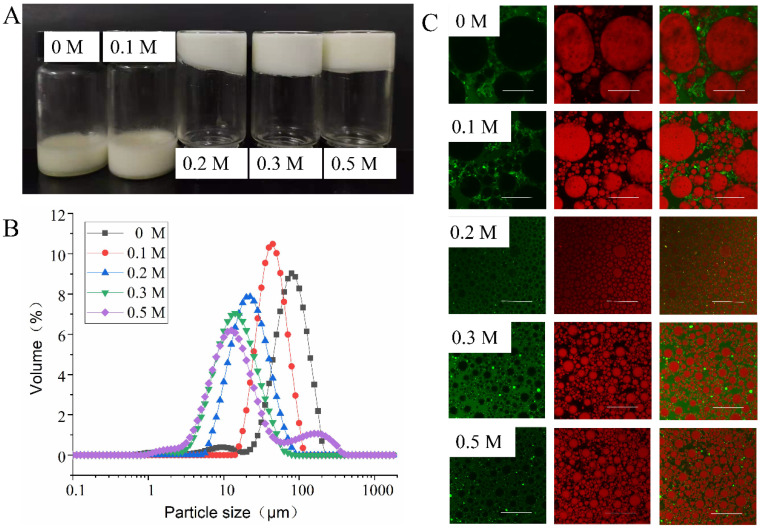
The visual observation (**A**), particle size distribution (**B**), and CLSM images (**C**) of HIPEs stabilized by EYGs at different NaCl ionic strengths. The concentration of EYGs was 3.0 wt%, and the volume of the oil phase was 0.8. The corresponding bar is 100 μm.

It could be seen in Figure 4A that at NaCl ionic strengths of 0 and 0.1 M, the HIPEs stabilized by EYGs presented a liquid-like concentrated emulsion. The HIPEs in liquid form were thought to be unstable for practical applications [9]. In contrast, at NaCl ionic strength over 0.1 M, HIPEs stabilized by EYGs appeared as gel-like emulsions that could hang upside down at the bottom of the bottle without slipping off. These phenomena reflected that EYGs at low NaCl ionic strength (<0.1 M) could not stabilize HIPEs, while they could at high NaCl ionic strength (>0.2 M). These results might be due to the increasing NaCl ionic strength, and the EYGs gradually dissociated from insoluble aggregates into smaller soluble micelles. In addition, the LDL and HDL obtained after dissociation were more easily adsorbed to the oil-water interface [21]. Previous studies also showed that disrupted EYGs by NaCl were more effective in forming and stabilizing oil-in-water emulsions than natural EYGs [24]. Moreover, high-charged particles failed to stabilize emulsions because of the “image charge” seen by them at the oil-water interface and encountered exclusive energy barriers as they approached the interface [25]. For highly charged EYGs (0 and 0.1 M), the convective forces that forced them to move to the interface during emulsification might not counteract the repulsive forces. This was why they had difficulty adsorbing to the oil-water interface, failing to form a stable emulsion. For example, HIPEs stabilized by gliadin colloidal particles with a zeta potential of +21.6 mV were also unstable [3].

It is well known that the size distribution of HIPEs is an important indicator of the performance of the emulsion. With the increasing NaCl ionic strength from 0 to 0.5 M, the particle size of the HIPEs stabilized by EYGs shifted to a smaller distribution, implying that the average droplet size of the emulsions was decreasing continuously. The HIPEs stabilized by EYGs at NaCl ionic strength of 0.5 M had the smallest droplet size (Figure 4B). According to previous reports, HIPEs with small particle sizes had relatively high stability [26]. The HIPEs stabilized by EYGs at NaCl ionic strength of 0 and 0.1 M had relatively large particle sizes, which might lead to the inability to form stable HIPEs. Similarly, with increasing NaCl ionic strength, the particle size of HIPEs stabilized by starch/whey protein isolate complex also decreased [9].

The microstructure of the emulsion, including the interfacial structure and the distribution state of the droplets, could be detected by CLSM, which was a key feature connected with the physical properties of Pickering HIPEs. In the fluorescence image, the oil droplets in the emulsion were dyed red by Nile red, while the proteins were dyed green by FITC. The fluorescence images of all the gel-like emulsions seemed to indicate that proteins formed thin barriers around the oil droplets, and the proteins in the aqueous phase might interact to act as steric hindrances around the oil droplets (Figure 4C). This result implied that EYGs at high NaCl ionic strengths (>0.2 M) could be well adsorbed to the oil-water interface and had the ability to form stable oil-in-water HIPEs. At NaCl ionic strengths above 0.2 M, the presence of network structure in the emulsion implied the formation of stronger emulsion gels [7], which was similar to the result of high viscoelasticity of HIPEs in rheological properties. Large droplets of HIPEs could be observed at low NaCl ionic strength (0 and 0.1 M), which was consistent with the results of particle size distribution. Moreover, it could be found that smaller EYGs were adsorbed to the oil-water interface with the NaCl ionic strength increased, which might be due to the dissociation of EYGs.

#### 3.3.2. Effect of pH on Emulsion Properties

**Figure 5:**
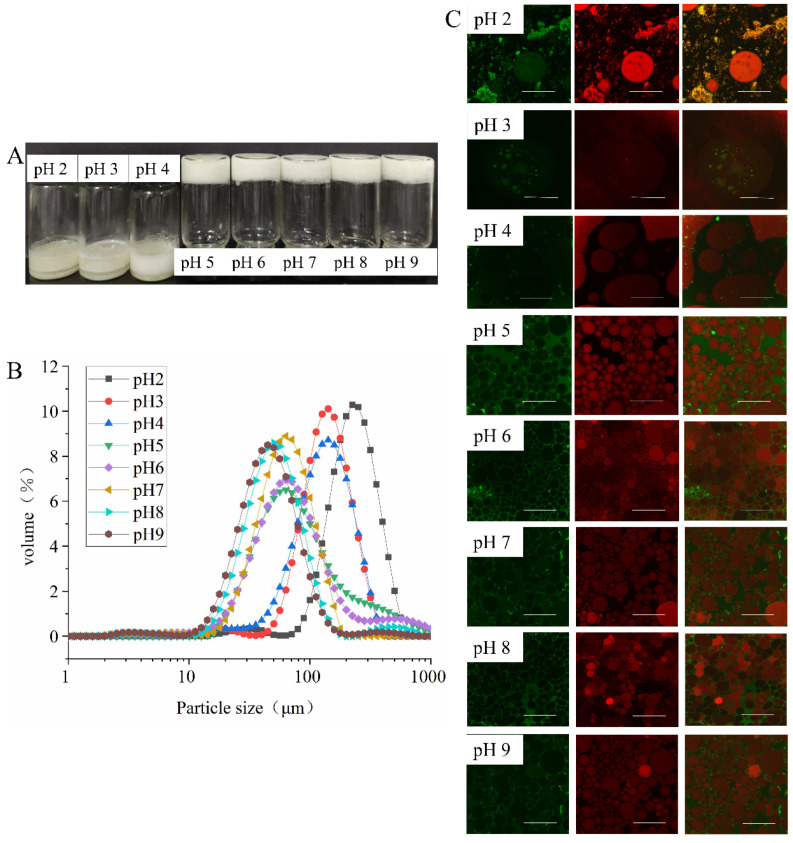
The visual observation (**A**), particle size distribution (**B**), and CLSM images (**C**) of HIPEs stabilized by EYGs at different pH. The concentration of EYGs was 3.0 wt%, the NaCl ionic strength was 0.3 M, and the volume of the oil phase was 0.8. The corresponding bar is 100 μm.

As shown in Figure 5A, the HIPEs stabilized at pH 2.0 to 4.0 were phase-separated liquid emulsions, which indicated that no stable Pickering emulsion system had formed. The oil droplets moved upward to form a gel layer, and the aqueous phase formed a serum layer at the bottom due to gravity. In contrast, HIPEs stabilized by EYGs at pH from 5.0 to 9.0 were in the form of gel-like emulsions that could hang upside down at the bottom of the bottle without slipping off. These phenomena indicated that regulation of pH could lead to solid-like or liquid-like states of HIPEs stabilized by EYGs. The reason for this result might be the fact that at NaCl ionic strength of 0.3 M, the EYGs continuously dissociated from large aggregates into smaller particles and micelles as the pH increased from 2.0 to 9.0 [14]. Therefore, at higher pH (5.0 to 9.0), smaller particle fractions and released phospholipid micelles could migrate and adsorb faster to the oil-water interface [20,27].

From the results of CLSM, at pH 5.0 to 9.0, the EYGs formed a continuous network structure in the aqueous phase, trapping the tightly packed oil droplets, which might be responsible for the formation of gel-like emulsions (Figure 5C). In addition, the relatively high surface charge at pH 2.0 and 3.0 might prevent the aggregation and adsorption of EYGs at the oil-water interface due to electrostatic repulsion. On the contrary, at pH over 4.0, the low surface charge enhanced the adsorption of EYGs at the oil-water interface. Moreover, previous studies found that the surface tension between oil and water in EYGs-stabilized emulsions decreased continuously with increasing pH at NaCl ionic strength of 0.15 M [20]. Under alkaline conditions, triglycerides were hydrolyzed to mono- and diglycerides, whose adsorption led to a decrease in oil-water interfacial tension [20].

As the pH increased from 2.0 to 9.0, the particle size of the emulsions became smaller (Figure 5B). Moreover, it was also observed in the results of CLSM that the particle size of the emulsion droplets was very large at pH from 2.0 to 4.0, while the particle size of the emulsion droplets was significantly reduced at pH from 5.0 to 9.0 (Figure 5C). With the increase in pH, the EYGs disintegrated. During emulsification, the high solubility and small size might cause the particles to adsorb faster at the new oil-water interface, thus showing the small oil droplets [20]. Therefore, emulsions prepared from EYGs under alkaline conditions had higher emulsifying stability than those under acidic conditions [28]. As mentioned above, mono- and diglycerides formed under alkaline conditions as small molecular surfactants might be helpful to the barrier formed around oil droplets. They could compete with EYGs for adsorption at the oil-water interface, which accelerate the decrease in surface tension and lead to smaller oil droplets [20]. Previous studies also found decreasing trend in particle size of emulsion stabilized by EYGs as the pH increased from 3.0 to 9.0 at NaCl ionic strength of 0.15 M [20]. In addition, among all gel-like emulsions, the particle size of the emulsion at pH 5.0 was the largest, which was similar to other researchers’ studies [15]. However, the decreasing trend of emulsion particle size as the pH value moved away from the isoelectric point (pH 5.0) was contrary to the study by other researchers [15]. This result might be attributed to the high oil content of the emulsion system or the effect of NaCl on the EYGs.

#### 3.3.3. Effect of Concentration of EYGs on Emulsion Properties

**Figure 6:**
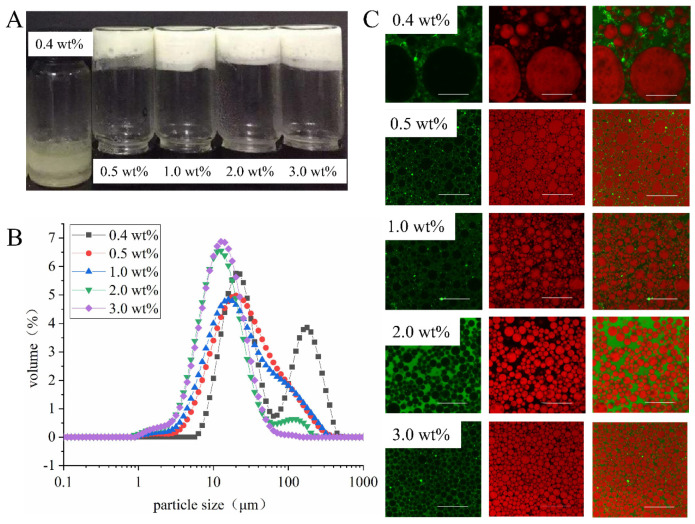
The visual observation (**A**), particle size distribution (**B**), and CLSM images (**C**) of HIPEs stabilized by EYGs at different concentrations. The NaCl ionic strength was 0.3 M, and the volume of the oil phase was 0.8. The corresponding bar is 100 μm.

As shown in Figure 6A, the HIPEs stabilized by EYGs at a concentration of 0.4 wt% were in the form of liquid-like emulsions with phase separation. However, they were solid-like appearances and did not flow after inversion with the addition of EYGs over 0.5 wt%. This phenomenon indicated that the minimum concentration required to form homogeneous and gelatinous HIPEs was 0.5 wt%. Moreover, the increase in the concentration of EYGs promoted the decrease in the particle size of the droplets, which improved the stability of HIPEs (Figure 6B). These phenomena might be due to that EYGs were loosely arranged at the oil-water interface and not enough to wrap all the oil phases at low concentration (<0.5 wt%) (Figure 6C) [15]. The droplets of emulsions were prone to agglomeration, resulting in extreme instability of HIPEs. Similarly, the HIPEs stabilized by soy β-conglycinin could only form gel-like emulsion at high concentration (>0.2 wt%) but present flow dynamics at low concentration (0.1 wt%) [29]. Furthermore, at high concentrations (>2.0 wt%), the oil droplets were closely packed with each other inside the emulsion, which might lead to the appearance of HIPEs resembling elastic gels [30].

#### 3.3.4. Effect of φ on Emulsion Properties

**Figure 7:**
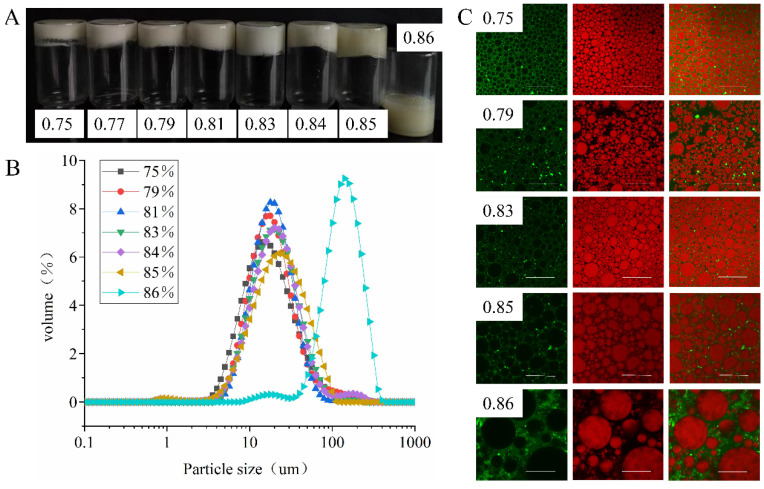
The visual observation (**A**), particle size distribution (**B**), and CLSM images (**C**) of HIPEs stabilized by EYGs with the different oil phases. The concentration of EYGs was 3.0 wt%, and the NaCl ionic strength was 0.3 M. The corresponding bar is 100 μm.

As shown in Figure 7A, the HIPEs stabilized by EYGs were able to form self-supporting emulsion gels at φ from 0.75 to 0.85. While at φ of 0.86, the HIPEs became liquid-like and flowed freely, which was probably due to the agglomeration among droplets, resulting in phase separation of the emulsion [5]. Previous studies showed that the increase in the internal phase ratio could reduce the stability of emulsion [29]. Because the oil droplets were subjected to repulsive forces from more surrounding droplets, facilitating droplet agglomeration and Ostwald ripening. In addition, as the φ increased, the oil droplets became larger, which was probably due to that the amount of EYGs particles per unit surface area adsorbed at the oil-water interface decreased [29]. The result of the particle size distribution of the HIPEs also proved that as φ continuously increased from 0.75 to 0.86, the particle size of HIPEs continued to increase (Figure 7B). As shown in the results of CLSM (Figure 7C), EYGs in the aqueous phase formed a continuous network through mutual interaction and trapped the oil droplets, which might be responsible for the formation of HIPE gels. While φ exceeded 0.83, the droplets of HIPEs underwent deformation due to dense packing, especially at φ of 0.85. Similarly, the HIPEs stabilized by egg chalaza also show the phase inversion with 86% oil content [7]. The accumulation and deformation of droplets into irregular shape was a distinct microstructural feature of HIPEs [31]. This result was also observed in HIPEs prepared from ovalbumin at φ of 0.91 [10].

#### 3.3.5. Rheological Properties of HIPEs

**Figure 8:**
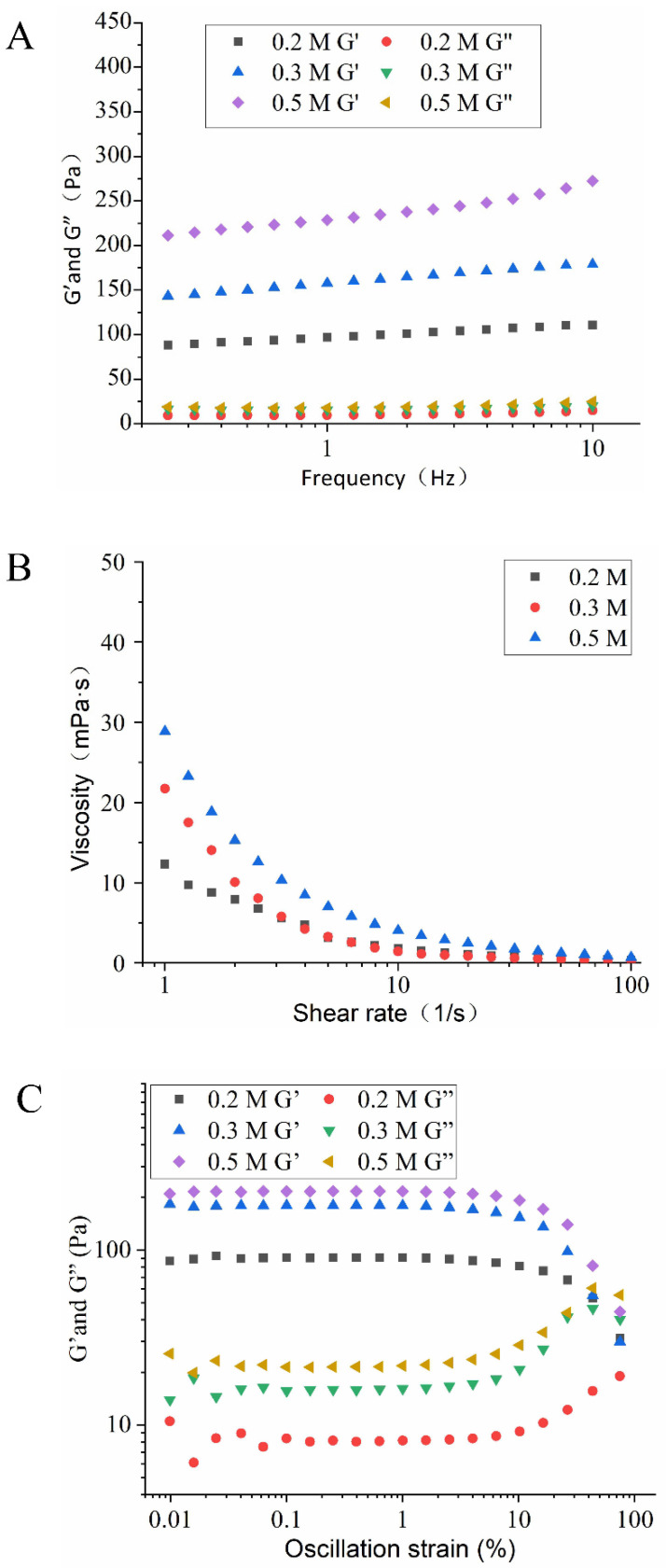
Rheological behavior of the HIPEs with 0.8 φ and 3.0 wt% EYGs at different NaCl ionic strength. Storage (G′) and loss (G″) moduli of the HIPEs as a function of frequency (**A**). Apparent viscosity of the HIPEs as a function of shear rate (**B**). G′ and G″ of the HIPEs as a function of oscillation strain (**C**).

**Figure 9:**
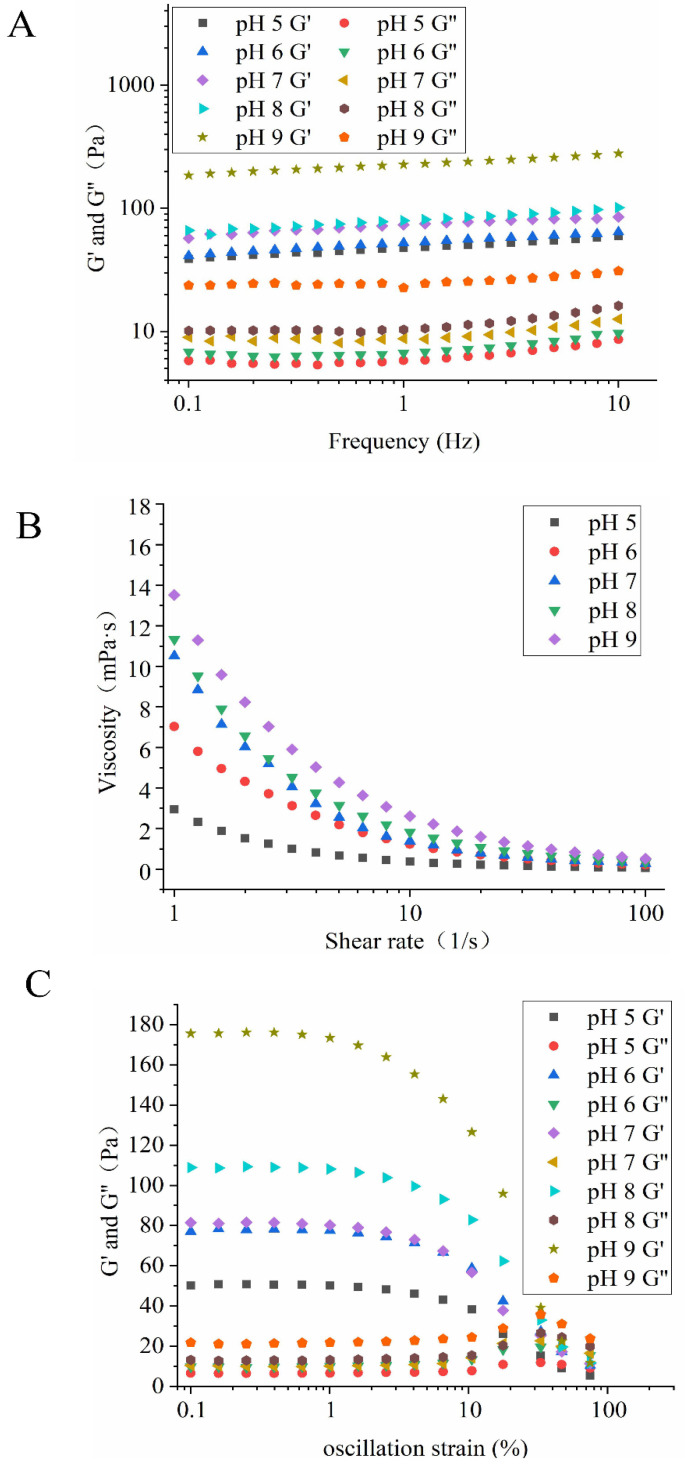
Rheological behavior of HIPEs stabilized by 3.0 wt% EYGs at NaCl ionic strength of 0.3 M with different pH. The φ is 0.8. Storage (G′) and loss (G″) moduli of the HIPEs as a function of frequency (**A**). Apparent viscosity of the HIPEs as a function of shear rate (**B**). G′ and G″ of the HIPEs as a function of oscillation strain (**C**).

Besides the size distribution and microstructure, the application and processing properties of HIPEs are strongly associated with their rheological properties [32]. The effect of NaCl ionic strength and pH on the rheological properties of HIPEs stabilized by EYGs could be seen in Figure 8 and Figure 9. The result of dynamic frequency sweep reflected that G′ was higher than G″ (Figure 8A and Figure 9A), which indicated that the HIPEs stabilized by EYGs were mainly elastic-like and exhibited strong gelatinous behavior. The formation of gelatinous HIPEs might be due to the tightly packed oil droplets [29], which was reflected in the results of CLSM (Figure 4C). Furthermore, the G′ value of HIPEs was gradually increased with the growing NaCl ionic strength (Figure 8A), which was opposite to the trend of particle size. This result might be due to that EYGs gradually dissociated into smaller soluble micelles with the increasing NaCl ionic strength, resulting in more EYGs adsorbed to the oil-water interface. Zhang et al. (2020) also found that an increased number of colloidal nanoparticles adsorbed at the oil-water interface could enhance the viscoelastic structure of HIPEs with the increasing NaCl ionic strength [33]. Meanwhile, the G′ value of HIPEs under alkaline conditions was higher than that under acidic conditions (Figure 8A). This result might also be attributed to the dissociation of EYGs in response to changes in pH. Similarly, it also had been reported that pH affected the interfacial packing behavior of zein particles and their cross-linking behavior in the aqueous phase [2]. Therefore, the network structure formed might be influenced, resulting in different viscoelasticity of HIPEs at different pH values (Figure 8B and Figure 9B).

With the shear rates increased, significant shear thinning behavior of the HIPEs could be observed (Figure 8B and Figure 9B), which might be due to that the interacting EYGs were destroyed by shear stress at high shear rates. In addition, the viscosity of emulsions rose gradually with increasing pH; correspondingly, the particle size of the oil droplets gradually became smaller. This result could be attributed to the fact that in a fixed volume of oil, the smaller oil droplets, the more friction points there were [20]. Aggregation of EYGs in the aqueous phase also contributed to friction among droplets and increased viscosity. Furthermore, the degree of decomposition of EYGs increased with the growing pH. Smaller EYGs particles were easier to adsorb to the oil-water interface. Therefore, the composition of barrier layers on the surface of the oil droplets might also be changed with pH, which might increase the interaction among the oil droplets and lead to higher viscoelasticity [20]. Moreover, the viscosity of emulsions rose gradually with increasing NaCl ionic strength, which was opposite to the trend of particle size. Similar results had also been presented in moringa seed residue protein [34].

The results of stress sweeps showed that G′ still dominates the rheological properties in HIPEs (Figure 8C and Figure 9C). This result suggested that the dominant elastic behavior and the stiffness of the gel-like HIPEs gradually improved for all samples. The values of G′ and G″ still showed an increasing trend with growing NaCl ionic strength and pH, which was similar to the results of frequency sweeps and strain sweeps. In addition, oscillatory yield stress (the stress value at the crossover point) could well reflect the flow performance of the HIPEs. Below the oscillatory yield stress, the HIPEs behaved as solid-like, dominated by elasticity. Above the oscillatory yield stress, the HIPEs structure was disrupted and transformed from solid-like to liquid-like, flowing like fluid [32]. In summary, the results of rheological properties further confirmed the results of CLSM and the visual appearance of HIPEs. The HIPEs stabilized by EYGs under suitable NaCl ionic strength and pH were viscoelastic and self-supporting. Therefore, the HIPEs stabilized by EYGs had great potential to substitute for PHOs in the food field [7].

#### 3.3.6. Storage Stability of HIPEs

**Figure 10:**
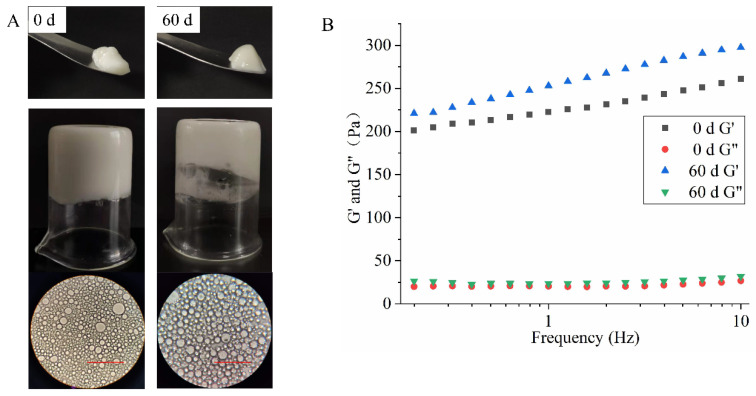
Visual appearance, microstructure (**A**), and frequency sweeps (**B**) of HIPEs stabilized by 3.0 wt% EYGs at NaCl ionic strength of 0.3 M before and after 60 days. The φ is 0.8. The corresponding bar is 100 μm.

As shown in Figure 10, HIPEs stabilized by 3.0 wt% EYGs at 0.3 M showed no significant changes in visual appearance and optical microscopy images after 60 days of storage and could hang upside down at the bottom of the bottle without slipping off. Meanwhile, oil leakage and creaming stratification could not be observed in the samples after 60 days of storage, which indicated that HIPEs stabilized by EYGs had suitable anti-emulsification and coalescence stability. However, in rheological tests, HIPEs showed an increase in G′ and G″ after 60 days of storage. This result might be due to the evaporation of water from the HIPEs during storage, which led to higher oil content, thus increasing the emulsion viscosity [26]. As a result, the migration of droplets slowed down, which might lead to the formation of stronger network structures and improve the stability of the HIPEs. Similarly, Li et al. (2020) found that HIPEs stabilized by meat protein particles formed stronger and more homogeneous network structures after 60 days of storage, which resulted in stronger viscoelasticity [35].

## 4. Conclusions

In this study, EYGs as potential emulsifiers of Pickering particles for stabilizing HIPEs were reported. HIPEs could be successfully prepared with an internal phase up to 85% and concentration of EYGs as low as 0.5 wt%, respectively, through a one-step homogenization method. At high NaCl ionic strengths (>0.2 M), the strong calcium phosphate of the EYGs was disrupted, causing the EYGs to decompose into small particles and micelles. At the NaCl ionic strength of 0.3 M, as the pH increased from 2.0 to 9.0, the EYGs disintegrated from the aggregated state into small particles and micelles. The smaller particles and micelles were more easily adsorbed at the oil-water interface, forming thin barriers around the oil droplets to prevent coalescence. Therefore, EYGs could stabilize self-supporting HIPEs at NaCl ionic strengths over 0.2 M and at pH over 5.0 with NaCl ionic strength of 0.3 M. Due to the irreversibility of EYGs as Pickering particles adsorbed at the oil-water interface, the HIPEs had suitable storage stability. In addition, the interacting EYGs formed a continuous network, and the droplets packed tightly, endowing the HIPEs with great viscoelasticity and self-supporting properties. The rheological results also showed that HIPEs stabilized by EYGs were able to convert liquid oil into solid gels. This study showed the potential of bioactive EYGs-stabilized HIPEs as replacements for PHOs in the food industry.

## Data Availability

Not applicable.
